# 3D Conformal Fabrication of Piezoceramic Films

**DOI:** 10.1002/advs.202106030

**Published:** 2022-04-28

**Authors:** Shiyuan Liu, Yao Shan, Ying Hong, Yuankai Jin, Weikang Lin, Zhuomin Zhang, Xiaote Xu, Zuankai Wang, Zhengbao Yang

**Affiliations:** ^1^ Department of Mechanical Engineering City University of Hong Kong Hong Kong China

**Keywords:** conformal ceramic films, energy harvesters, structural health monitoring, tactile sensor arrays, vibration sensors

## Abstract

Piezoceramic films are an essential class of energy‐conversion materials that have been widely used in the electronics industry. Although current methods create a great freedom for fabricating high‐quality piezoceramic films, it requires well‐controlled synthesis conditions, including special high‐cost equipment and planar substrates particularly. The limited substrate selections hinder the applications of piezoceramic films in 3D conformal structures where most objects possess complex curvilinear surfaces. To overcome such limitations, a fast, energy‐efficient, and cost‐effective approach, named flame treated spray (FTS) coating, is developed for preparing piezoceramic films on free‐form surfaces. The flame treatment significantly enhances the hydrophilicity of a substrate, assisting in forming a uniform and continuous thin film. The followed spray coating deposits hundreds of nanometers to several micrometers thick films on 3D free‐form surfaces. Given the size controllability and arbitrary surface compatibility of the FTS method, a highly conformal piezoelectric tactile sensor array (4 × 4) is assembled on a spherical surface for mimicking robot fingers and an on‐site thin‐film sensor on the wing of an aircraft model to monitor the vibration in real‐time during flight. The FTS film deposition offers a highly promising methodology for the application of functional thin‐film from micro‐ to marcoscale devices, regardless of conformal problems.

## Graphical abstract

Functional ceramic films are available to be manufactured on freeform substrates for demanding applications via a facile flame treatment assisted spray coating process. The developed technique enables the conformal coating of piezoceramic thin films on various targets, including robotics, airplanes, etc.

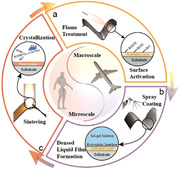

## 1 Introduction

Piezoceramics, especially piezoceramic thin films, are emerging materials receiving a great deal of interest in recent decades owing to their unique mechanical strength, dielectric properties, and energy conversion characteristics.^[^ ^1^, ^2^ ^]^ Modern manufacturing technologies, including chemical solution‐based synthesis,^[^ ^3^ ^]^ chemical vapor deposition,^[^ ^4^ ^]^ magnetron sputtering,^[^ ^5^ ^]^ and pulsed laser deposition,^[^ ^6^ ^]^ have fabricated high‐performance piezoelectric thin films for applications in energy harvesters, sensors, and actuators.^[^ ^7^, ^8^, ^9^ ^]^ Currently, the inherently planar piezo thin films fabrication technologies require rigid and flat substrates such as silica and glasses materials and highly clean laboratory environments.^[^ ^10^ ^]^ The rigorous fabrication conditions confine the piezoelectric thin films to 2D forms,^[^ ^11^ ^]^ posing immense obstacles in utilizing piezoelectric thin films for 3D conformal structures, such as artificial skins, body joints, and even large equipment like aircraft coatings.^[^ ^12^ ^]^

The chemical solution‐based technology, especially the sol–gel process, has been regarded as the preferred cost‐effective approach to massively produce thin films.^[^ ^13^ ^]^ For large‐area thin films, spray coating is more suitable than spin or dip coating since there are no limitations on the size or geometry of the substrate.^[^ ^14^ ^]^ To assist the film forming process, the precursor for spray coating usually contains sol‐solution and powders, and thereafter, the derived films are formed by accumulated particles and droplets from the spray gun.^[^ ^15^ ^]^ Note that the addition of powders will increase the thickness of films. It, however, is accompanied by the raised porosity that cannot meet requirements for some demanding applications.^[^ ^7^, ^16^ ^]^ Furthermore, when the droplets cannot form a uniform and dense liquid film on the substrate, a great deal of cracks will be produced during the sintering process. Therefore, thin films fabricated by spray coating usually require well‐designed equipment to precisely control the droplets and film formation.^[^ ^14^ ^]^

Herein, for the first time, we introduce a modified sol–gel based flame treated spray (FTS) coating method to fabricate conformal ceramic thin films on substrates with 3D free‐form surfaces in a fast, energy‐efficient, and cost‐effective manner. By sweeping the flame across the surface of the material, we generate a series of functional groups on the substrate surface. The introduction of the flame treatment significantly improves the hydrophilicity, solving the natural issue that sprayed dropwise solution is hard to form a uniform liquid film on original substrates. Following that, the sol–gel solution wets the surface easily and spreads to form a dense film without cracks and pores. It is worth noting that the residues on the material surface after the flame treatment can be removed by a high‐temperature sintering process or paper wiping. The fast flame treatment does not influence the intrinsic properties of the substrate material or the fabricated film quality.

By applying the FTS coating method, we successfully fabricate conformal piezoceramic thin films on free‐form surfaces of metal foils. Due to the high thermal stability, stainless steel is mainly used as the substrate for thin film growth. In this study, we propose to adopt the low‐cost FTS method to provide a universal fabricating route for high‐quality Ba_0.85_Ca_0.15_Zr_0.1_Ti_0.9_O_3_ (BCZT) thin films, an emerging BaTiO_3_‐based material with good piezoelectric performances,^[^ ^17^, ^18^ ^]^ on freeform stainless steel substrates. The compositional elements of BCZT are commonly seen in many fields and the production cost of BCZT is much lower than other materials. The nontoxic characteristic of BCZT also provides a potential application on implant devices like artificial limbs for multiple purposes. We comprehensively investigate the energy harvesting performances of BCZT thin films grown on flat, concave, and convex surfaces of stainless steel. A 4 × 4 piezoelectric tactile sensor array is successfully assembled on a spherical shell that manifests the application potential in robotics in a microscale. To show the feasibility of directly coating piezoceramic thin film on complex equipment, we fabricate the BCZT thin film on the wing of an airplane model as a vibration sensor to monitor the flying fluctuations.^[^ ^19^ ^]^ We further successfully manufactured different ceramic thin films on different free‐form substrate materials and demonstrate the broad compatibility of the FTS method.

## 2 Results

### 2.1 Design and Processing Route

**Figure 1:**
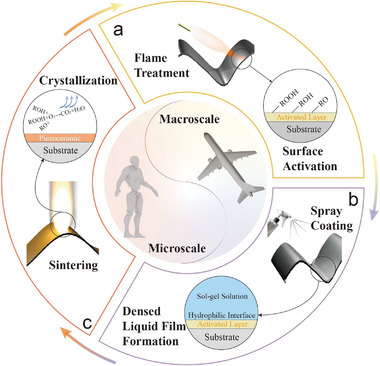
Processing flow of conformal thin film fabrication on complex substrates. a) The flame treatment process and the schematic of the improvement of surface energy. b) Spray coating of liquid thin films onto as‐treated substrate. c) Thin film sintering process for sintering. The overall procedures can be repeated for multiple cycles to achieve the target thickness.

The formation of dense and uniform thin film by the sol–gel technique requires a high wettability of the target substrates. The commonly used surface energy boosting methods, including corona and plasma treatments, need complex equipment and are confined in a small treatment area, limiting the film coating on large and 3D free‐form surfaces. To address the issue, we here present a fast and cost‐effective approach named flame treated spraying (FTS) coating. The overall film‐fabrication strategy is shown in **Figure** **1**. After cleaning the substrate, the surface energy is boosted by the flame generated by a commonly used lighter, as shown in Figure 1a. The combustion process contains a series of chemical reactions, and the primary reaction occurs between oxidant (oxygen in the atmosphere) and hydrocarbon (butane in this work). The reaction terminates by producing vast amounts of CO_2_, H_2_O, and heat. Before the complete combustion reaction, some active radical species are produced, which can be concluded as the following chemical reaction (1)^[^ ^20^ ^]^

(1) $$\mathrm{RH}+O_{2}\to \left\{\begin{array}{c} R^{\cdot }+{\;}^{\cdot }\mathrm{OOH}\\ RO^{\cdot }+{\;}^{\cdot }\mathrm{OH} \end{array}\to \to CO_{2}+H_{2}O+\mathrm{Heat}\right.$$

The active species produced by the incomplete combustion remains on the surface of the substrate material under the action of flame jet. The fast flame treatment process only needs less than one second to significantly improve the wettability. Long‐time treatment is not necessary since it may destroy the substrate because of the high‐temperature deformation and oxidation reactions of substrates. The functional area is controlled by the flame size and the sweeping path.

As shown in Figure 1b, we use the spray coating to deposit the sol–gel precursor solution to i) fabricate large‐scale thin films and ii) adapt to various substrates with arbitrary morphologies. Assisted by the active radical species generated from the flame treatment, the precursor uniformly wets the surface and form a dense liquid film, which is essential to the following sintering process, as shown in Figure 1c. The residual radical species will react with oxygen in the air during the sintering process and the gel film will crystalize at a high temperature. The thickness of the obtained film relies on the concentration of the sol–gel precursor and the repeated film coating cycles as depicted in Figure 1.

**Figure 2:**
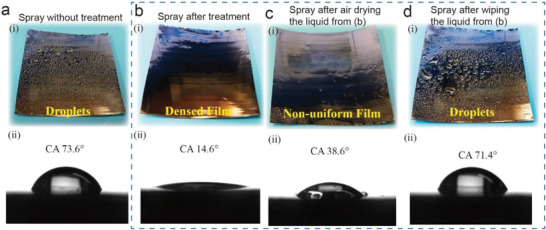
Optical photos of droplet film formation after spray coating and the related contact angle. a) The liquid sol solution directly sprays on the substrate without any pretreatment. b) The liquid sol solution sprays on the substrate after the flame treatment. c) The liquid sol solution sprays on the substrate after drying the liquid film from b) by air. d) The liquid sol solution sprays on the substrate after drying the liquid film from b) by paper wiping.

To further examine the influence of flame treatment on the wettability of the substrates, we compare the spreading state and contact angle of precursor solution on the stainless steel foil slices (with the size of 0.01 × 50 × 50 mm) before and after treatments, as illustrated in **Figure** **2**. Without any treatment on the surface of stainless steel, the droplets sprayed on the material are totally dispersive, see Figure 2a(i). Such dropwise liquid would not form a uniform thin film after the drying and sintering process. To quantify the wettability, we use a goniometer to measure the static contact angle of water droplet (200 µL in this work) on the substrate. In Figure 2a(ii), the contact angle is 73.6°, which is not good enough for the liquid to wet the surface entirely. To improve the wettability of the substrate, the flame from a commonly seen jet lighter is applied to quickly sweep the generated flame across the surface of the stainless steel slice. When spraying the liquid on the flame‐treated surface, almost all the dispersed droplets merge to form a complete and uniform liquid film and the contact angle is decreased to 14.6°, as shown in Figure 2b. Such a filmwise liquid state facilitates the formation of the crystallized solid film during the sintering process. To further investigate the effectiveness of hydrophilicity optimization, the substrate with as‐formed liquid film is dried by airflow and resprayed the liquid. As shown in Figure 2c(i), most droplets wet the surface, however, the liquid film is not as continuous as before. Some holes are observed on the surface, and the contact angle is measured as 38.6°, indicating that the active radical species are partially removed by water evaporation. If the drying procedure is replaced by paper wiping, as depicted in Figure 2d, almost all the active radical species are removed and the sprayed droplets on the substrate return to the dropwise liquid as nontreated substrate. According to the Young–Dupré equation

(2) $$W_{\mathrm{SLG}}=\gamma_{\mathrm{LG}}\left(1+\cos\theta \right)$$

where W_SLG_ is the surface energy (or liquid adhesion energy), *γ*_LG_ is the interfacial energy of liquid and gas, and *θ* is the contact angle. In our case, *γ*_LG_ is a constant, and W_SLG_ is increasing when the *θ* is reduced. Therefore, the higher hydrophilicity improved by the flame is accompanied by the higher surface energy.

To verify the existence of the active radical species, we use Fourier transform infrared spectroscopy (FTIR) to characterize the surface of the stainless steel substrate, as shown in Figure S1 (Supporting Information). Comparing to the nontreated substrate, the treated substrate shows an intense transmittance signal near 2600 cm^−1^ indicating the residual CO_2_ molecules. It can be seen that a weak peak appears near 3500 cm^−1^ representing the existence of OH bonding. The air‐dried sample shows weaker peaks in both 3500 and 2600 cm^−1^, and the paper‐wiped sample barely shows the characteristic peaks indicating that the active radical species can be easily removed and the fast flame treatment will not bring side reaction with the surface of the material. We also demonstrate the generality of the flame treatment for improving the surface hydrophilicity of different materials, such as glass, mica, and Si wafer. As shown in the following Figure S2 (Supporting Information), the results show that the flame treatment process improves the hydrophilicity of each material: glass (52.9°–14.0°), mica (35.1°–7.0°), and Si wafer (86.9°–5.1°).

### 2.2 Fundamental Characterization

**Figure 3:**
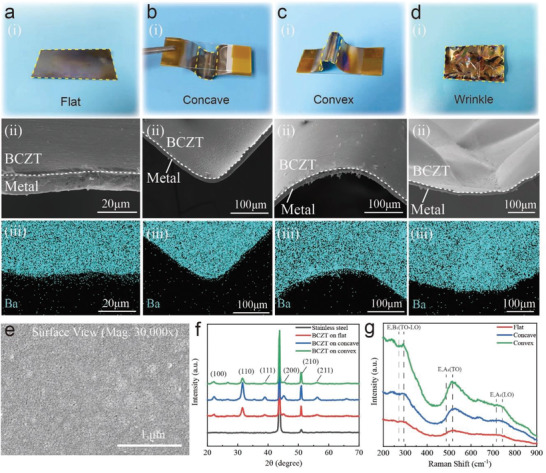
Materials characterizations of thin films grown on 3D free‐form surfaces. Thin films grown on the flat surface a), concave surface b), convex surface c), and arbitrarily wrinkled surface d), respectively. The subtitled images show the i) The optical image of the prototype. ii) The SEM cross‐sectional image of the thin film. iii) The EDS result of the Ba element distribution. e) The surface morphology of the BCZT thin film with 30 000x magnification. f) The XRD results of the BCZT thin films grown on flat, concave, and convex surfaces. g) The Raman results of the BCZT thin films grown no flat, concave, and convex surfaces.

Although PZT is the most widely used piezoelectric material, considering its toxicity and the strict restrictions on lead‐containing materials raised by RoHS, we in this study use lead‐free Ba_0.85_Ca_0.15_Zr_0.1_Ti_0.9_O_3_ (BCZT) ceramics. BCZT has been verified to have a piezoelectric performance comparable to that of PZT.^[^ ^21^ ^]^ To demonstrate the feasibility of the FTS method on 3D free‐form surfaces, we bent a 0.01 mm thick stainless steel foil and formed flat, concave, convex, and arbitrary wrinkled surfaces. After air‐drying and fast sintering processes, piezoceramic thin films are successfully fabricated on 3D free‐form surfaces, as shown in **Figure** **3**a(i)–d(i). The film thickness applied in the following tests is ≈1 µm obtained by 2 cycles of the FTS coating as shown in Figure S3b (Supporting Information). The minimum and maximum film thicknesses we successfully manufactured are ≈500 nm and ≈8 µm as shown in Figure S3a–c (Supporting Information). The SEM cross‐sectional images in Figure 3a(ii)–d(ii) show the film morphology grown on each type of surface. According to the SEM images, the crack‐free, uniform, and continuous piezoceramic thin films are successfully grown on 3D free‐form surfaces. For the wrinkled surface, the substrate contains various sharp angles. It can be seen that the piezoceramic thin film can still grow along with such sharp angles without appearing apparent cracks. It manifests that BCZT thin films are uniformly grown on 3D free‐form surfaces through the EDS mapping results as the uniform distribution of the Ba element shown in Figure 3a(iii)–d(iii). The elements distribution of Ba, Ca, Zr, and Ti are shown in Figure S4 (Supporting Information). Owing to the fact that the background contains Zr element to some extent, it seems that the concentration of Zr element is close to Ba element. Since the whole process of the FTS method is processed in air, it inevitably leaves impurities on the surface of the film. The magnified surface morphology from the thin film grown on the concave surface is also examined in Figure 3e. Apart from several impurities on the surface, the entire thin film is dense and uniform. To examine the surface roughness of the thin films, we use atomic force microscope (AFM) to scan the surfaces of the film in tapping mode, as shown in Figure S6 (Supporting Information). The root mean square roughnesses (Rq) for thin films grown on flat, concave, and convex surfaces are 26.8, 13.0, and 26.4 nm, indicating that the films attained via the developed FTS method have low roughness.

To further examine the crystallinity of the thin films grown on 3D free‐form surfaces, Figure 3f shows the X‐ray diffraction (XRD) pattern of the BCZT thin films grown on flat, concave, and convex surfaces. The typical characteristic peaks of perovskite structure for the BCZT material and stainless steel substrate can be, respectively, observed from the XRD patterns. The Raman spectrum in Figure 3g further verifies the crystal structure of the BCZT thin film. Each thin film shows three typical scattering bands, including B_1_, E(TO+LO) near 300 cm^−1^, E, A_1_(TO) near 520 cm^−1^, and E, A_1_(LO) near 720 cm^−1^ relating to the tetragonal phase.

### 2.3 Piezoelectric Performances

**Figure 4:**
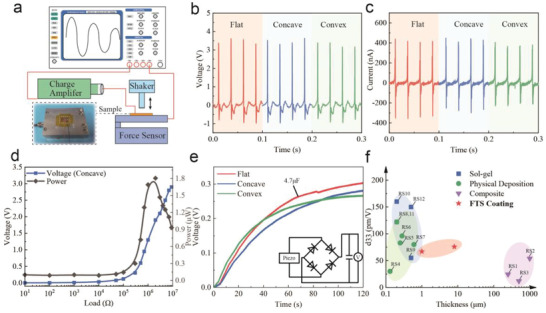
Piezoelectric performances of the BCZT thin films grown on 3D free‐form surfaces. a) The experiment setup for the piezoelectric performance characterization. b) The open‐circuit voltage output of the three types of films under tapping stimulation. c) The short‐circuit current output of the three types of films under tapping stimulation. d) The impedance matching result for the BCZT thin film grown on the concave surface. e) The capacitor charging capability of three types of thin films. f) The films thicknesses and d_33_ of flexible BCZT‐based piezoelectric materials summarized from the published paper. RS indicates the references in the supporting information.

The electrical output of the thin films grown on 3D free‐form surfaces was measured and stimulated by a periodically compressive force generated by an electrodynamic shaker, as shown in **Figure** **4**a. To eliminate the measuring error brought by the irregular morphology of different samples, the samples are cut into 1 × 1 cm slices and fixed on the force sensor. The epitaxial growth of the films ensures that no peeling off by mechanical forces will not happen during the testing period. Owing to the high conductivity of stainless steel, no extra bottom electrode layer needs to be fabricated before testing. The conductive Cu tape is used as the top electrode to assemble a simple piezoelectric nanogenerator (PENG). The oscilloscope, current preamplifier and charge amplifier are connected to the PENG to measure the open‐circuit output voltage, short‐circuit current, and open‐circuit charge, respectively.

By adjusting the frequency of the shaker and the distance from the hammer to the PENG, the compressive force is fixed to 4 N, where the frequency is 40 Hz. Under such stimulation, as shown in Figure 4b, the average output voltages of thin films grown on flat, concave, and convex surfaces are 3.3, 3.4, and 3.1 V, respectively. Figure 4c shows the corresponding average output currents as 420, 425, and 395 nA, respectively. The piezoelectric coefficient d_33_ is one of the critical parameters to estimate the relationship between the external force change and the charge generation. In this work, we use piezoresponse force microscopy (PFM) to measure the d_33_ of BCZT thin films grown on flat, concave, and convex substrates to compare the piezoelectric performance with the existed work related to BCZT material. The detailed measurement and calculation processes have been described in the Experimental Section; and Figure S9 (Supporting Information). The value of d_33_ reaches 66–76 pm V^−1^ for each type of BCZT thin film. Therefore, the BCZT thin films grown on 3D free‐form surfaces via the FTS method have similar piezoelectric performances as those grown on a traditional flat surface.

The impedance matching experiment further estimates the instantaneous power of the PENGs under different external load resistance. The voltage output keeps increasing as the load resistance rises from 10 Ω to 10 MΩ. For the PENG fabricated on a concave surface, the maximum instantaneous power reaches 1.8 µW, as shown in Figure 4e. The flat PENG shows a maximum of 1.9 µW and the convex one shows 1.8 µW, as shown in Figure S7 (Supporting Information). As shown in the inlet of Figure 4f, a full‐bridge rectifier is adopted to rectify the alternating current from the PENG and store energy in a capacitor of 4.7 µF. In Figure 4e, the capacitor can be charged to ≈0.3V in 120 s by each type of PENG. The results show that the BCZT film fabricated via the FTS method has comparable piezoelectric performances, as listed in Figure 4f; and Table S1 (Supporting Information).

**Figure 5:**
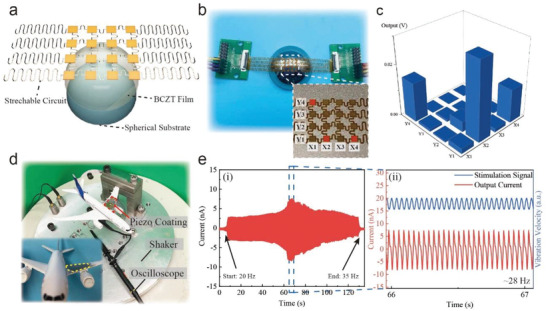
The assembled piezoelectric tactile sensor array for mimicking the robot fingers and the vibration sensor for aircraft structural health monitoring. a) The layout of the piezoelectric tactile sensor array on the spherical surface. b) The setup of the tactile sensor array. Each electrode pad is connected to one port for data collection, respectively. c) The data output of the sensor when the spontaneous forces are applied on X1Y4, X2Y1, X4Y1 pads. d) The setup of the vibration experiment and the inlet image show the complex surface and the film coating position. e,i) The piezoelectric output under the vibration within the frequency range 20–35 Hz and ii) The zoom‐in signal around 28 Hz vibration under the sinusoidal stimulation.

**Figure 6:**
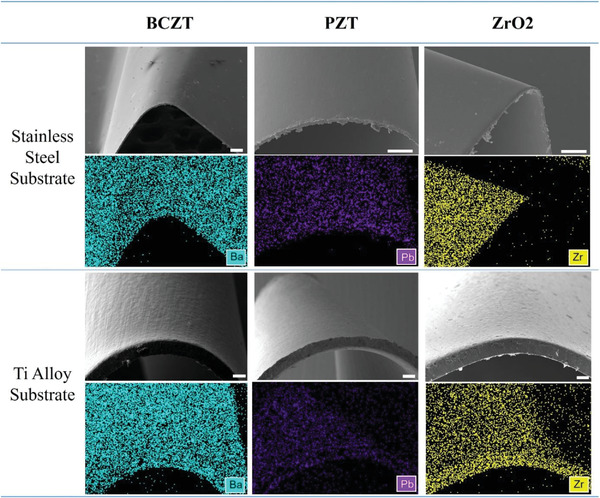
The capability of thin films grown on 3D free‐form surfaces. a–c) The BCZT, PZT, and ZrO_2_ thin films grow on stainless steel with a convex surface, respectively. d,e) The BCZT, PZT, and ZrO_2_ thin films grow on Ti alloy with a convex surface, respectively. All the scale bars are 100 µm.

When introducing piezoceramic films into flexible electronics, researchers usually design microstructures to attain a conformal contact to curved 3D host structures.^[^ ^22^ ^]^ Though a high‐performance conformal system can be obtained after a series of complicated MEMS processing, the film is still difficult to totally fit the surfaces of nonzero Gaussian curvatures, such as spherical and hyperboloidal surfaces. Here, we successfully deposit the BCZT thin film onto a spherical surface, mimicking the topology of a fingertip, and integrate it with stretchable and conformal electrodes as a piezoelectric tactile sensor array. The layout of the sensor array is shown in **Figure** **5**a. The SEM images of the BCZT thin film grown on spherical surface are shown in Figure S5 (Supporting Information). The thin film shows highly uniform and crack‐free on the spherical surface, which demonstrates the feasibility of FTS method on nondevelopable objects. The spherical substrate is conductive stainless steel, which is also served as the negative electrode and ground connection. A thin layer of BCZT thin film is deposited onto the spherical surface. An electrode array with 4 × 4 electrode pads covers the BCZT film surface. As shown in Figure 5b, each electrode pad can transmit the generated signal to the oscilloscope via an independent wire. **Figure** **6**c displays the instantaneous signal amplitude obtained by tapping three points (X1Y4, X2Y1, X4Y1) simultaneously. The signal records of all sensors within 1 s are illustrated in Figure S10 (Supporting Information). This result shows that the piezoelectric film prepared by the FTS method can be directly integrated with the existing sensor assembling technologies and applied to robotic systems.

Taking advantage of the electromechanical coupling effect, piezoceramics have been utilized in nondestructive testing sensors for detecting abnormal vibration conditions of engineering assets in real‐time.^[^ ^23^ ^]^ Though the bulky sensors comprised of piezoelectric wafers or composites have shown promising capabilities in structural health monitoring, installing external devices will increases the burden and breaks the integrity of the equipment due to their heavy weights and fixed geometries.^[^ ^12^ ^]^ To overcome the limitations, in this study, facilitated by the FTS process, we directly fabricate the piezoceramic thin film (BCZT) onto the curved surface of airplane wing to show the potential application of on‐site structural health monitoring. The mechanical vibration of the airplane wing wave can be detected as electric signals in real‐time functioned by the deposited piezoceramic thin film. As schematically shown in Figure 5d, the FTS‐fabricated piezoceramic film works as a vibration sensor that carefully deposited on the surface of one edge of the wing. In Figure 5e, the measuring system consists of a vibration shaker to generate vibration input and an oscilloscope to record the output signal (see the Experimental Section for detailed experiment setup). Figure 5e(i) shows the recorded current signal, from which we recognize that the hazardous vibration occurs at around 65 seconds (the corresponding current is around 8 nA) on the frequency of around 28 Hz. To further understand the vibration behavior at hazardous conditions, we present the detailed input vibration (blue line) and output current (red line) signal at the frequency of 28 Hz in Figure 5e(ii). The results manifest the feasibility of depositing light‐weight piezoelectric thin‐film‐based sensors on complex equipment surfaces with high integrity for real‐time structural health monitoring.

### 2.4 Generality

To verify the generality and capability, we implement the FTS method on different ceramic thin films and substrates. In this study, we mainly focus on the nontoxic piezoelectric material BCZT thin films grown on the most commonly used stainless steel substrate foil, as shown in Figure 6a. We also successfully fabricate PZT thin film, another widely used high‐performance piezoceramic material, on the convex surface as illustrated in Figure 6b. The SEM and EDS results verify that the PZT thin film uniformly covers the convex surface. Apart from the piezoelectric ceramic, the FTS coating strategy has been manifested to manufacture other thin‐film materials. In this study, we deposit the ZrO_2_ ceramic thin film, a widely used structural ceramic material with high mechanical strength, via the FTS method on the convex stainless steel surface and examined by SEM and EDS in Figure 6c. Furthermore, the substrate material is not only limited to stainless steel, but the alternative Ti alloy, a widely used metal with high‐temperature sustainability, is also demonstrated to grow ceramic thin films in this study. The SEM results of the BCZT, PZT, and ZrO_2_ thin films grown on 0.1 mm thick Ti alloy with the convex surface are shown in Figure 6d–f. No apparent cracks are found on the film surfaces indicating that the thin films are successfully deposited on 3D free‐form surfaces via the FTS method.

The typical features of FTS coating and several current standard film preparation methods are listed in **Table** **1**. Physical thin film deposition methods, especially magnetron sputtering and pulsed laser deposition, can precisely control the film growth quality and obtain films with micropatterns through templates. However, limited by the apparatus and substrate specifications, physical deposition methods are challenging to fabricate large‐area thin films. For chemical processes like spin coating and tape casting that have been applied in industry, they are challenging to prepare thin films on surfaces of arbitrary morphologies. The FTS coating strategy overcomes the issues mentioned above and we believe it will provide new insight into the academic and industry of thin‐film materials.

**Table 1:** The comparison between the FTS coating and other current thin film fabrication technologies

	Methodology	Film crystalline	Economic efficiency^a)^	Applicable scenarios	Film geometry	Scale
Physical method	Magnetron sputteirng^[^ ^24^ ^]^	Single & polycrystal	Low	Laboratory	Flat, micropattern	Wafer size
	Pulsed Laser depostion^[^ ^25^ ^]^	Single & polycrystal	Low	Laboratory	Flat, micropattern	Wafer size
Chemical method	Tape casting^[^ ^26^ ^]^	Polycrystal	Medium	Laboratory, factory	Flat	Wafer size
	Spin coating^[^ ^27^, ^28^ ^]^	Single & polycrystal	Medium	Laborotary, factory	Flat	Wafer size
	FTS (this work)	Polycrystal	High	Laboratory, factory	Free‐form	Arbitrary Size

^a)^ The economic efficiency is considered based on the expenses on the apparatus, operation difficulty, and environmental requirements.

## 3 Discussion and Conclusion

By applying flame treatment, the substrate material will obtain high hydrophilicity that is beneficial for sol solution to wet the surface and form a continuous and uniform thin film. The FTS method allows fabricating thin films on surfaces with arbitrary morphologies efficiently and economically. We mainly demonstrate the manufacturing of the lead‐free material BCZT thin film on stainless steel foil with 3D free‐form surfaces. The material characterization results verified the good crystallinity of the thin film, and the energy harvesting performances prove the application potential on functional coatings. For most thin‐film based work on piezoelectric energy harvesting, the microelectromechanical systems (MEMS) process is inevitable for assembling high‐performance devices, which also brings complicated operations and high costs. Since our proposed FTS process is a facile method to prepare thin films on freeform substrates, we also hope that the subsequent device preparation process is also low‐cost and simple, so as to provide a technical basis for industrial‐scale preparation. However, the simple device fabrication process may bring some side effects, such as device packaging problems, durability issues, etc., which need to be improved by further systematic optimization. Finally, we demonstrate preparing piezoelectric thin films on the spherical shell as conformal tactile sensor arrays and on airplane models as a vibration sensor to show the potential of the FTS method to directly deposit functional layers in various complex equipment and environments in the future.

## 4 Experimental Section

### Preparation of Thin‐Film Sol–Gel Solution— BCZT Sol–Gel Solution

4.1

The BCZT precursors, including barium acetate (TCI), calcium acetate (TCI), titanium isopropoxide (Alfa Aesar, 98%), and zirconium n‐propoxide (TCI, 70% in 1‐proponol) were stoichiometrically mixed and dissolved in acetic acid (TCI) based on the formula of Ba_0.85_Ca_0.15_Zr_0.1_Ti_0.9_O_3_. The solution was diluted to 0.3 m by 2‐methoxyethanol (Alfa Aesar, 99%) and distilled water to improve the stability. The as‐prepared solution was stored in a sealed container at room temperature for at least 3 days aging before use.

#### 4.2 Preparation of Thin‐Film Sol–Gel Solution—PZT Sol–Gel Solution

The PZT precursors, including lead acetate trihydrate (TCI), titanium isopropoxide (Alfa Aesar, 98%), and zirconium n‐propoxide (TCI, 70% in 1‐proponol) were stoichiometrically mixed and dissolved in 2‐methoxyethanol (Alfa Aesar, 99%) based on the formula of PbZr_0.52_Ti_0.48_O_3_. The solution was diluted to 0.3 m by 2‐methoxyethanol and distilled water to improve the stability. The as‐prepared solution was stored in a sealed container at room temperature for at least 3 days aging before use.

#### 4.3 Preparation of Thin‐Film Sol–Gel Solution—ZrO_2_ Sol–Gel Solution

The ZrO_2_ precursor, including zirconium n‐propoxide (TCI, 70% in 1‐proponol) is dissolved into 2‐methoxyethanol (Alfa Aesar, 99%) and dilute to 0.3 m by 2‐methoxyethanol. The solution maintains stability in a sealed vessel for 1–2 days at room temperature.

### 4.1 Thin Film Coating on 3D Free‐Form Surfaces

The substrates mainly used in this study are Ni‐Cr metal thin foil and Ti‐alloy metal foil purchased from Dongguan Ruipu Metal Industry. The metal is arbitrarily bent and fixed on a glass substrate by two ends to obtain the 3D free‐form surfaces.

To improve the hydrophilicity of the substrate, the flame treatment was implemented. By using a commonly seen jet lighter, the flame was quickly swept over the surface of the substrate to improve the surface energy. It is worth noting that the residence time of the flame on the surface of the substrates be short enough to avoid any chemical reaction or thermal deformation occurring on the materials.

After the flame treatment, the liquid sol was sprayed onto the 3D free‐form surfaces to form a dense and uniform liquid film. For some extremely sharp morphologies, spraying only may be difficult to cover the surface uniformly. In this case, a roller or swap was implemented to wipe the surface to make the film evenly. The liquid film was dried by hot air and followed by rapid sintering at 800 ℃.

### 4.2 Materials Characterizations

The cross‐sectional images and top‐surface view images were examined by scanning electron microscopy (SEM, FEI Quanta 450). The element distribution was examined by the energy dispersion spectroscopy (EDS, Oxford Instruments, INCA Energy 200). The contact angles were measured by Standard Contact Angle Goniometer (OCA25, Dataphysics). The crystal structures were characterized by X‐ray diffraction (XRD, Rigaku SmartLab). The Raman spectrum of the thin films was characterized by Raman spectrometer (Perkinelmer Raman station 400F). The FTIR spectrum was conducted by Fourier transform infrared spectrometer (Bruker Tensor 27).

### 4.3 Piezoelectric Performances Characterization

After the film coating and sintering process, the BCZT thin films were polarized under a corona electric field (5 kV cm^−1^) at room temperature for 30 min. To demonstrate the simplicity and ease of use of FTS method derived films, the sample was cut into 1 × 1 cm square pieces and directly paste the copper foil tape onto the surface of the BCZT film. Thus, the copper foil tape served as the top electrode and the stainless steel served as the bottom electrode, and they were welded by conductive wires. The piezoelectric performance tests were conducted by compressing the piezoelectric thin film‐based devices under a fixed frequency and force by a vibration generator. The open‐circuit voltage output was recorded by a digital oscilloscope (Rohde & Schwarz RTE1024) and the short‐circuit current was measured by a current preamplifier (Stanford Research SR570). The surface roughness and topology characterization was conducted by an atomic force microscope (Bruker Dimension Icon System). The conductive Co/Cr‐coated silicon probes (MESP‐RC‐V2, Bruker) were used in the piezoresponse force microscopy (PFM) test, with a nominal spring constant 5 N m^−1^ and a free‐air resonance frequency of ≈150 kHz. An AC drive voltage was applied on the sample at the resonance frequency of 685 kHz in contact mode.

### 4.4 Piezoelectric Tactile Sensor Array Test

In this study, a spherical shell made of stainless steel was used as the film deposition substrate. One thin layer of BCZT film was conformally coated onto the shell via the FTS method. The serpentine electrode array was purchased from Shenzhen Xingjie Electronic Co., Ltd, where the curved connection wires were obtained by laser cutting polyimide film, and the electrode pads were made of copper film and the upper and lower surfaces were connected to each other. The serpentine electrode array was physically contacted the BCZT thin film surface on the spherical shell. To ensure the close contact, the electrode array was prestretched and pressed onto the spherical surface. The thermoplastic polyurethane was used to encapsulate the system under 200 ℃ with prestretching and pressing forces. After encapsulation, the electrode array was tightly contacted with the BCZT film on the spherical shell. Then the stainless shell will serve as one electrode and the electrode array serves as another electrode. The data of each sensor pixel were recorded by the oscilloscope.

### 4.5 Aircraft Vibration Test

The aircraft vibration test was conducted by mounting an airplane model on an electromagnetic shaker (ETS Solutions (China) Ltd, L215M). The coated area was (5 ×  5 mm) on the wing of the aircraft model. The vibration acceleration rate was set as 0.5 g (acceleration of gravity). The aircraft model was fixed on the shaker via a steel clamp. In the vibration test, the vibration frequency was increased from 20 to 35 Hz with a rate of 7 Hz s^−1^. A Laser Vibrometer (Polytec NLV‐2500) was used to record the velocity response and the oscilloscope connected with the preamplifier was used to record the current output from the piezoelectric film.

### 4.6 Statistical Analysis

Data were mainly obtained by a digital oscilloscope (Rohde & Schwarz RTE1024) and the charge preamplifier (Stanford Research SR570). Data processing was operated by the Origin software (OriginLab).

## Conflict of Interest

The authors declare no conflict of interest.

## Supporting information

Supporting Information

## Data Availability

The data that support the findings of this study are available in the Supporting Information of this article.
