# Supplementary material for: 3D Conformal Fabrication of Piezoceramic Films
Source: Adv Sci (Weinh). 2022 Apr 28;9(18):2106030. doi: 10.1002/advs.202106030 (PMC9218746; doi:10.1002/advs.202106030)
Supplement: Supplementary file 1 — Supporting Information [file ADVS-9-2106030-s001.pdf]

Supporting Information

# 3D Conformal Fabrication of Piezoceramic Films

*Shiyuan Liu[1 †], Yao Shan[1 †], Ying Hong[1], Yuankai Jin[1], Weikang Lin[1], Zhuomin Zhang[1], Xiaote Xu[1], Zuankai Wang[1], Zhengbao Yang[1*]*

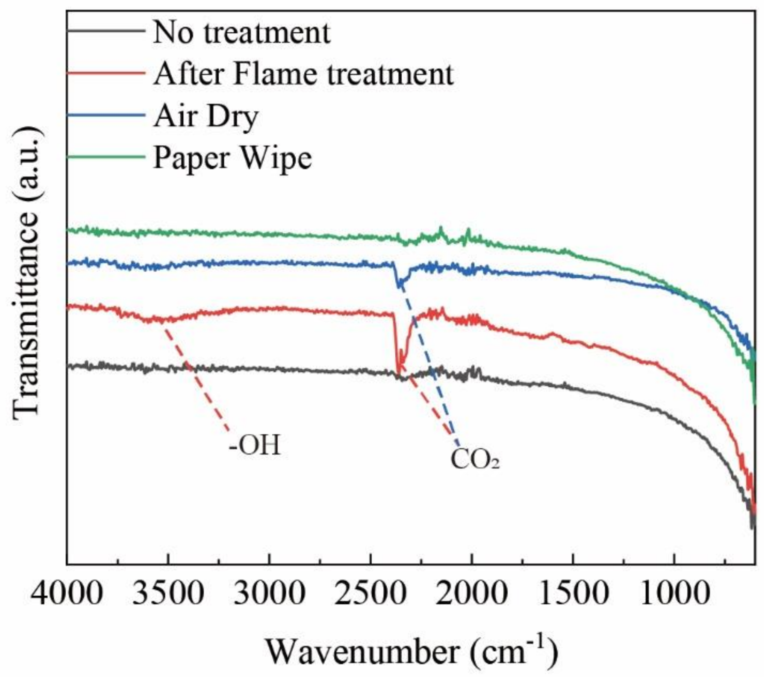


**Figure S1.** FTIR spectrum of the surface of the stainless steel foil before and after flame treatment.

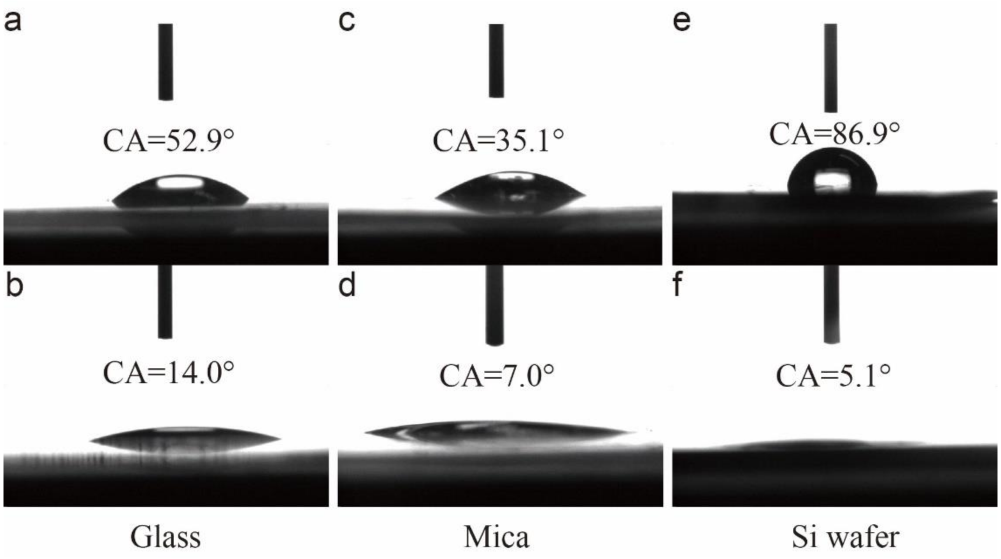


**Figure S2. The contact angle improvements by flame treatment.** (a-b) The glass, (c-d) the mica, and (e-f) the Si wafer.

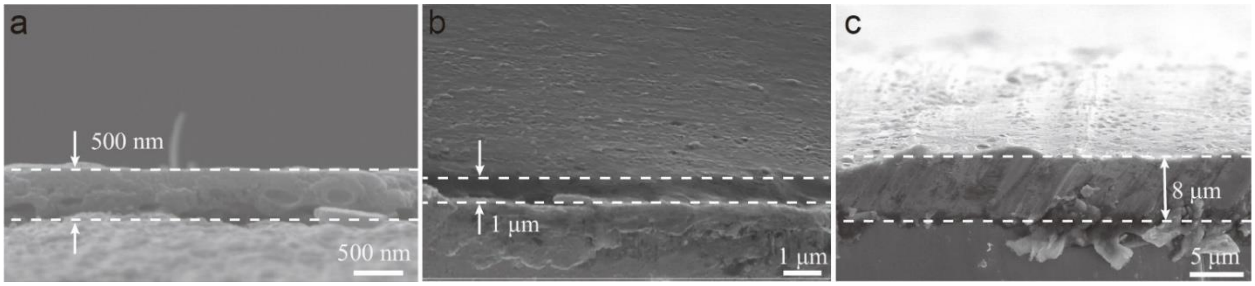


**Figure S3. Thickness range of piezoceramic thin films prepared by FTS method.** (a) The minimum thickness (~ 500 nm) was obtained in this work. (a) The thickness utilized adopted in piezoelectric performances tests in this study. (b) The maximum thickness (~ 8 μm) was obtained in this work.

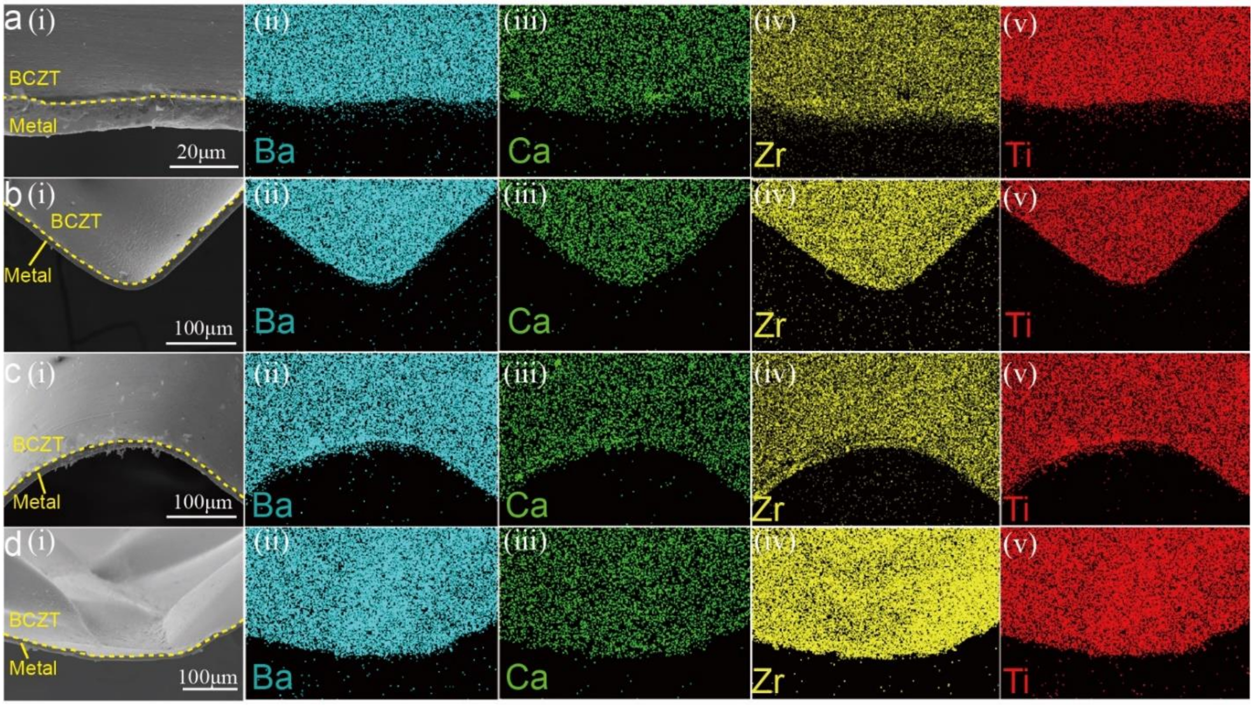


**Figure S4. The SEM and EDS characterizations of BCZT thin films grown on stainless steel with 3D free-form surfaces.** (a) The BCZT thin film grows on a flat surface. (b) The BCZT thin film grows on a concave surface. (c) The BCZT thin film grows on a convex surface. (d) The BCZT thin film grows on a wrinkled surface. The subtitled (ii)-(v) shows the Ba, Ca, Zr, and Ti on-site elements mapping results, respectively.

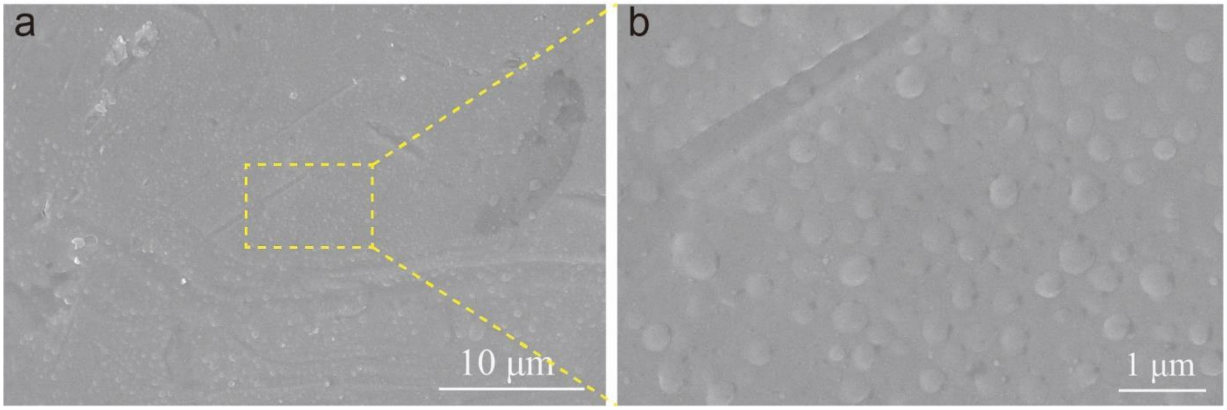

**Figure S5. The SEM characterization.** (a) The top surface of BCZT thin film coated on the spherical object. (b) The zoom-in view of the selected area in (a).

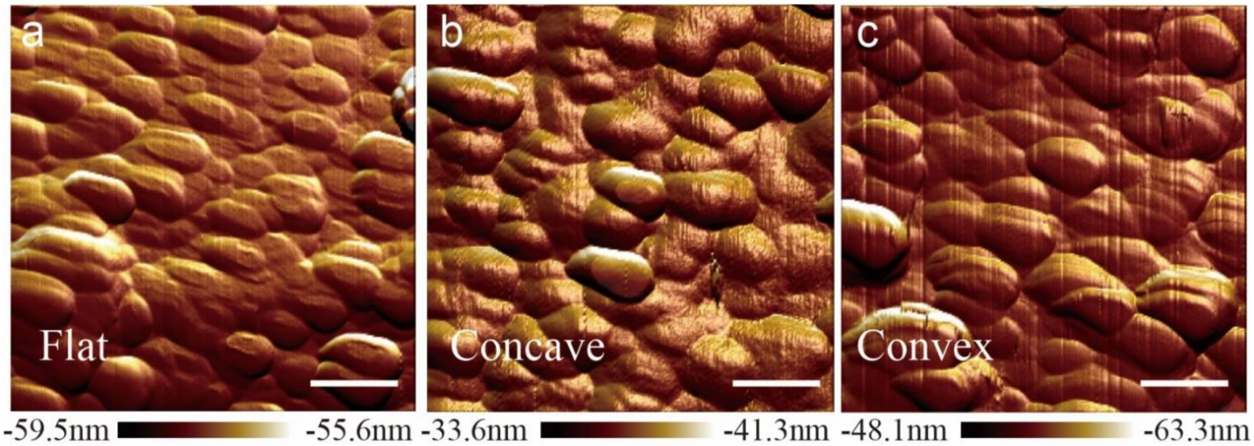


**Figure S6. The AFM characterization results of BCZT thin films grown on 3D free-form surfaces.** (a) The thin film grows on a flat surface. (b) The thin film grows on a concave surface. (c) The thin film grows on a convex surface.

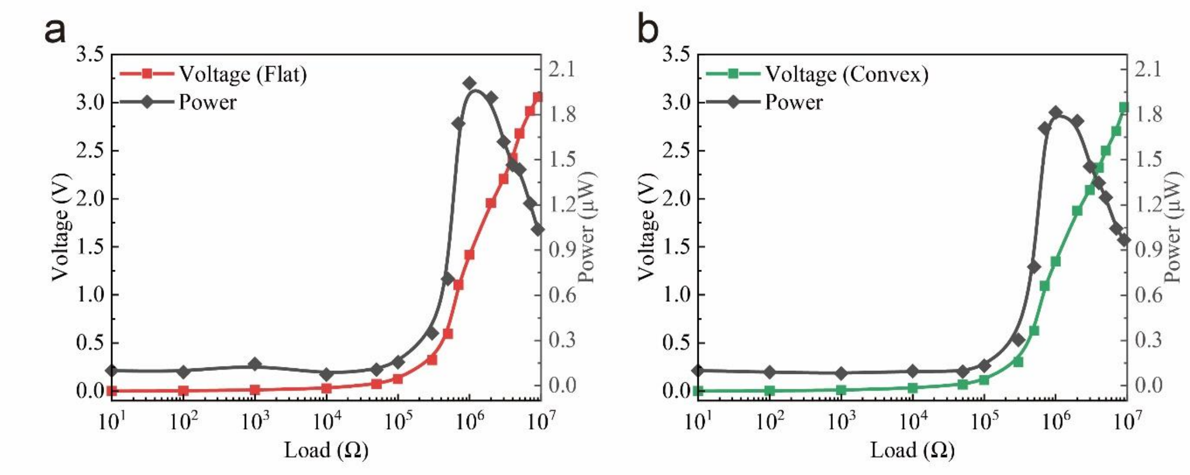


**Figure S7. The impedance matching tests results.** (a) The BCZT-based PENG is fabricated on a flat surface. (b) The BCZT-based PENG is fabricated on the convex surface.

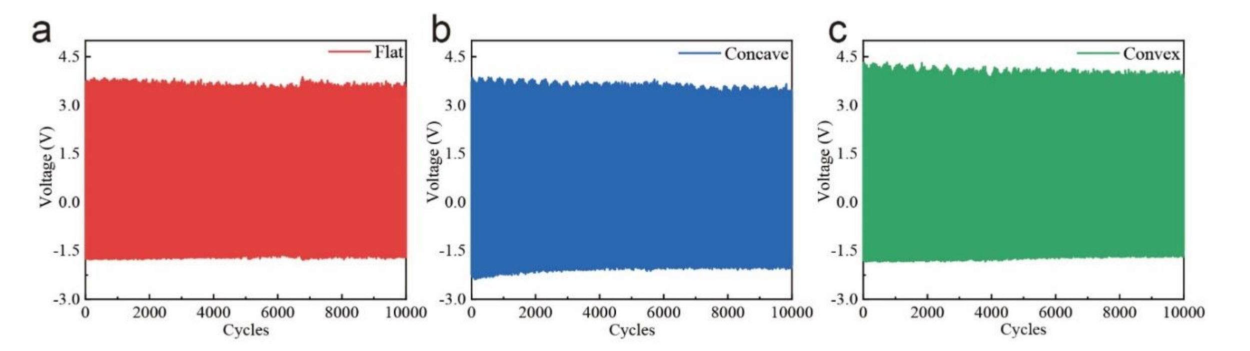


**Figure S8. The compression fatigue test results.** (a) The BCZT-based PENG was fabricated on flat surface. (b) The BCZT-based PENG was fabricated on the concave surface. (c) The BCZT-based PENG was fabricated on the convex surface.

Under the PFM test, the value of $d_{33}$ can be evaluated by

$$d_{33}=\frac{s}{Q_a V_{ac}},$$

where s is the piezoelectric response displacement, $Q_a$ (~ 5) is the average quality factor and $V_{ac}$ is the drive voltage applied on the sample. The average piezoresponse displacement of BCZT thin films on flat, concave, and convex surfaces are 331 pm, 381 pm, and 337 pm, respectively. The calculated average $d_{33}$ of BCZT films on three types of substrates are 66.2 pm/V, 76.2 pm/V, and 67.4 pm/V.

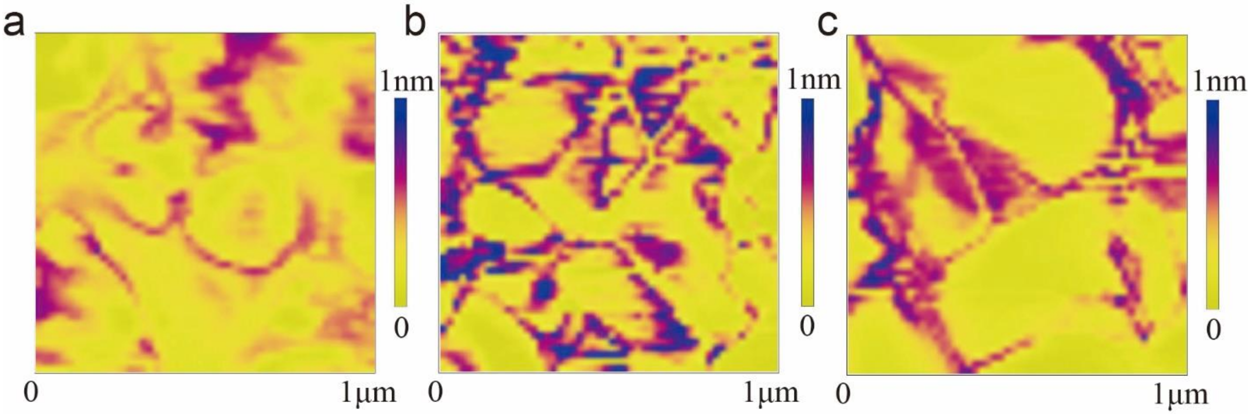


**Figure S9. PFM mapping of BCZT thin films (with the area of 1×1μm) on (a) flat, (b) concave, and (c) convex substrates.**

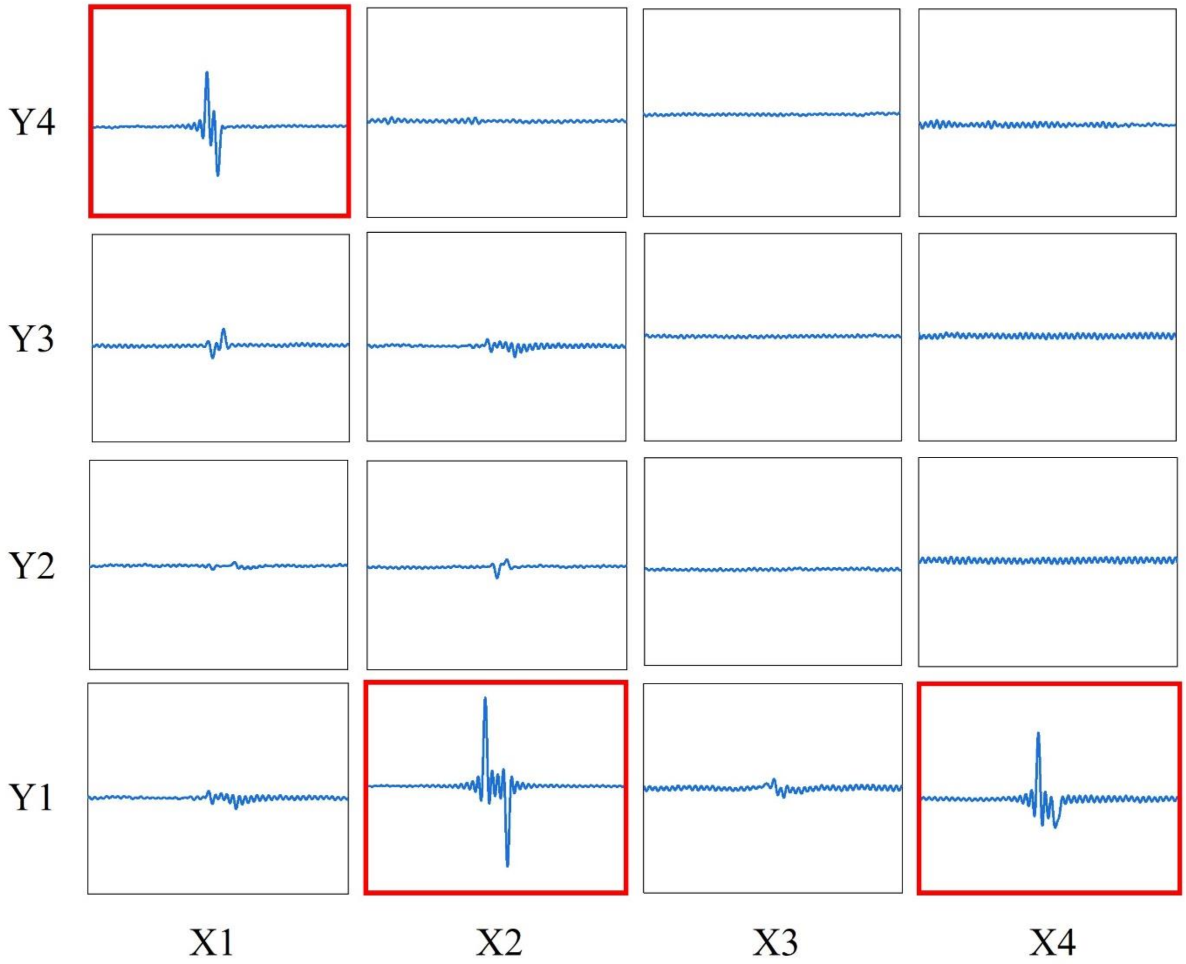


**Figure S10. Data recording of the 4×4 piezoelectric tactile sensor in one second.**

**Table S1.** The piezoelectric properties of BCZT-based PENGs fabricated via different techniques.

| Material | Method[a] | Substrate | Thickness (μm) | $d_{33}$ (pC/N) | Power density ($\mu W/cm^2$) | Reference |
|---|---|---|---|---|---|---|
| **Nanoparticle** | Hydrothermal | ITO | 250 | 11-38 | 6.4 | (*1*) |
| **Nanoparticle** | Hydrothermal | PDMS | 1000 | 54 | 2.6 | (*2*) |
| **Nanoparticle** | Solid Process | PDMS | 500 | 2-12 | - | (*3*) |
| **Thin film** | PLD | $IrO_2$/Si | 0.13 | 30 | - | (*4*) |
| **Thin film** | PLD | $SrTiO_3$ | 0.25 | 63-103 | - | (*5*) |
| **Thin film** | PLD | $SrTiO_3$ | 0.28 | 96 | - | (*6*) |
| **Thin film** | PLD | Pt/Ti/$SiO_2$/Si | 0.6 | 80 | - | (*7*) |
| **Thin film** | rf-Sputtering | Pt/Ti/$SiO_2$/Si | 0.2 | 122 | - | (*8*) |
| **Thin film** | Sol-gel | Pt/Ti/$SiO_2$/Si | 0.46 | 40-70 | - | (*9*) |
| **Thin film** | Sol-gel | Pt/Ti/$SiO_2$/Si | 0.2 | 90-230 | - | (*10*) |
| **Thin film** | Sol-gel | Pt/Ti/$SiO_2$/Si | 0.2 | 113-131 | | (*11*) |
| **Thin film** | Sol-gel | Mica | 0.5 | 150 | **0.9** | (*12*) |
| **Thin film** | **FTS** | **Metal alloy** | **1-8** | **66-76** | **1.8** | **This work** |

[a] This describes the method used for manufacturing BCZT material

Reference

1. H. Lu, H. Shi, G. Chen, Y. Wu, J. Zhang, L. Yang, Y. Zhang, H. Zheng, High-Performance Flexible Piezoelectric Nanogenerator Based on Specific 3D Nano BCZT@Ag Hetero-Structure Design for the Application of Self-Powered Wireless Sensor System. *Small*. **n/a**, 2101333 (2021).
2. Y. Zhang, C. K. Jeong, T. Yang, H. Sun, L.-Q. Chen, S. Zhang, W. Chen, Q. Wang, Bioinspired elastic piezoelectric composites for high-performance mechanical energy harvesting. *J. Mater. Chem. A*. **6**, 14546–14552 (2018).
3. X. Gao, M. Zheng, X. Yan, J. Fu, M. Zhu, Y. Hou, The alignment of BCZT particles in PDMS boosts the sensitivity and cycling reliability of a flexible piezoelectric touch sensor. *J. Mater. Chem. C*. **7**, 961–967 (2019).
4. C. J. M. Daumont, Q. Simon, E. Le Mouellic, S. Payan, P. Gardes, P. Poveda, B. Negulescu, M. Maglione, J. Wolfman, Tunability, dielectric, and piezoelectric properties of Ba(1−x)CaxTi(1−y)ZryO3 ferroelectric thin films. *J. Appl. Phys.* **119**, 94107 (2016).
5. Q. R. Lin, S. Li, D. Y. Wang, Built-in electric field in compositionally graded (1 − x)Ba(Zr0.2Ti0.8)O3–x(Ba0.7Ca0.3)TiO3 thin films. *RSC Adv.* **5**, 55453–55457 (2015).
6. Q. Lin, D. Wang, S. Li, Strong Effect of Oxygen Partial Pressure on Electrical Properties of 0.5Ba(Zr0.2Ti0.8)O3–0.5(Ba0.7Ca0.3)TiO3 Thin Films. *J. Am. Ceram. Soc.* **98**, 2094–2098 (2015).
7. A. Piorra, A. Petraru, H. Kohlstedt, M. Wuttig, E. Quandt, Piezoelectric properties of 0.5(Ba0.7Ca0.3TiO3) – 0.5[Ba(Zr0.2Ti0.8)O3] ferroelectric lead-free laser deposited thin films. *J. Appl. Phys.* **109**, 104101 (2011).
8. W. L. Li, T. D. Zhang, Y. F. Hou, Y. Zhao, D. Xu, W. P. Cao, W. D. Fei, Giant piezoelectric properties of BZT–0.5BCT thin films induced by nanodomain structure. *RSC Adv.* **4**, 56933–56937 (2014).
9. X. Jiang, D. Wang, M. Sun, N. Zheng, S. Jia, H. Liu, D. Zhang, W. Li, Microstructure and electric properties of BCZT thin films with seed layers. *RSC Adv.* **7**, 49962–49968 (2017).
10. S. R. Reddy, V. V. Bhanu Prasad, S. Bysakh, V. Shanker, J. Joardar, S. K. Roy, Ferroelectric and piezoelectric properties of Ba0.85Ca0.15Ti0.90Zr0.10O3 films in 200 nm thickness range. *J. Am. Ceram. Soc.* **102**, 1277–1286 (2019).
11. W. L. Li, T. D. Zhang, D. Xu, Y. F. Hou, W. P. Cao, W. D. Fei, LaNiO3 seed layer induced enhancement of piezoelectric properties in (100)-oriented (1−x)BZT–xBCT thin films. *J. Eur. Ceram. Soc.* **35**, 2041–2049 (2015).
12. S. Liu, Z. Zhang, Y. Shan, Y. Hong, F. Farooqui, F. S. Lam, W.-H. Liao, Z. Wang, Z. Yang, A flexible and lead-free BCZT thin film nanogenerator for biocompatible energy harvesting. *Mater. Chem. Front.* **5**, 4682–4689 (2021).
